# Analysis of risk factors for pre-diabetes to diabetes among cadres in Huzhou: A 5-year retrospective study

**DOI:** 10.5937/jomb0-57846

**Published:** 2026-01-06

**Authors:** Yan Wu, Jun Yao, Shitong Xing, Ying Zhang, Yan Shen

**Affiliations:** 1 Nanjing Medical University, Nanjing, 211166, China; 2 The First People's Hospital of Huzhou, The First Affiliated Hospital of Huzhou Teachers College, Huzhou, 313000, China

**Keywords:** diabetes, pre-diabetes, cadres' health care population, risk factors, dijabetes, predijabetes, zdravstvena zaštita zdravstvenih radnika, faktori rizika

## Abstract

**Background:**

To analyse the risk factors of Diabetes Mellitus (DM) through a five-year retrospective study of pre-diabetes mellitus (Pre DM) cadres.

**Methods:**

The cadres who underwent physical examination and were diagnosed with pre-diabetes in the First People's Hospital of Huzhou City from April 2019 to November 2024 were selected as the research objects, and their basic information (age, gender, body mass index, etc.), lifestyle (diet, exercise, smoking and drinking, etc.), family medical history, biochemical indicators (blood sugar, blood lipid, blood pressure, etc.) and other data were collected. People were divided into two groups based on whether they developed diabetes, and the risk factors for diabetes were determined using univariate analysis and multivariate Logistic regression analysis.

**Results:**

A total of 174 Pre DM cadres were included, and 30 of them developed diabetes. Univariate analysis revealed significant differences in age, body mass index, fasting blood glucose, triglycerides, uric acid, blood pressure, blood lipids, liver function, and renal function between the two groups (P&lt; 0.05). The results of the multivariate Logistic regression analysis showed that age, BMI, SBP TG, TC, BUN, TBIL, and ALT were independent risk factors for the development of diabetes in the pre-diabetic healthcare population within 5 years (P&lt; 0.05).

**Conclusions:**

This study demonstrates that age, BMI, blood pressure, blood lipid levels, liver and kidney function indexes, and gender are significant risk factors for patients with Pre DM to develop into DM. Monitoring and managing these factors can reduce the risk of pre-diabetes patients progressing to diabetes. This provides personalised health management suggestions for pre-diabetes (Pre DM) cadres and healthcare professionals and also offers theoretical support for the formulation of relevant health policies.

## Abstract (sr)

**Uvod:**

Analizirani su faktori za dijabetes melitus (DM) kroz petogodišnju retrospektivnu studiju medicinskog kadra sa predijabetesom (Pre DM).

**Metode:**

Istraživanjem je obuhvaćeno osoblje koje je prošlo sistematski pregled i kod kojeg je dijagnostikovan predijabetes u Prvoj narodnoj bolnici grada Hužou, u periodu od aprila 2019. do novembra 2024. Prikupljeni su podaci o osnovnim karakteristikama (godine starosti, pol, indeks telesne mase itd.), životnim navikama (ishrana, fizička aktivnost, pušenje i konzumacija alkohola), porodičnoj anamnezi, biohemijskim parametrima (nivo šećera u krvi, lipidi u krvi, krvni pritisak itd.) i drugim relevantnim informacijama. Učesnici su podeljeni u dve grupe - oni koji su razvili dijabetes i oni koji nisu. Faktori rizika za razvoj dijabetesa određeni su pomoću jednovarijantne analize i multivarijantne logističke regresione analize.

**Rezultati:**

U istraživanje je uključeno ukupno 174 osoba sa predijabetesom, od kojih je 30 razvilo dijabetes. Jednovarijantna analiza pokazala je statistički značajne razlike u godinama starosti, indeksu telesne mase, nivou glukoze u krvi na prazan stomak, trigliceridima, mokraćnoj kiselini, krvnom pritisku, lipidima u krvi, funkciji jetre i funkciji bubrega između dve grupe (P&lt; 0.05). Rezultati multivarijantne logističke regresione analize pokazali su da su godine starosti, BMI, sistolni krvni pritisak (SBP), trigliceridi (TG), ukupni holesterol (TC), urea (BUN), ukupni bilirubin (TBIL) i alanin aminotransferaza (ALT) nezavisni faktori rizika za razvoj dijabetesa kod populacije sa predijabetesom u roku od 5 godina (P&lt; 0.05).

**Zaključak:**

Ova studija pokazuje da su godine starosti, BMI, krvni pritisak, nivoi lipida u krvi, pokazatelji funkcije jetre i bubrega, kao i pol, značajni faktori rizika za prelazak iz predijabetesa u dijabetes. Pracenje i upravljanje ovim faktorima može smanjiti rizik od progresije predijabetesa u dijabetes. Studija pruža personalizovane smernice za upravljanje zdravstvenim kadrom sa predijabetesom, kao i teorijsku osnovu za kreiranje relevantnih zdravstvenih politika.

## Introduction

Diabetes mellitus (DM) is an endocrine and metabolic disease characterised by a chronic increase in blood sugar levels, which is caused by the weakening of islet function or insulin resistance resulting from various pathogenic factors. Type 2 diabetes mellitus, T2DM) is the main part of the diabetic population, accounting for more than 90%, and the main patients are middle-aged and older people. The main reason for the illness is that the body is not sensitive to insulin. The other type is type 1 diabetes mellitus (T1DM). Patients with this type of diabetes mellitus are primarily children and adolescents. The primary reason for this condition is the lack of insulin secretion resulting from an abnormal immune system [1] [2]. With the development of the economy and the change in lifestyle, the prevalence of DM is increasing year by year, which seriously threatens people's health and quality of life [3]. It has become a significant public health concern worldwide. According to data from the International Diabetes Federation (IDF), approximately 537 million adults worldwide had diabetes in 2021, and this number is expected to increase to 783 million by 2045 [4]. A large number of studies show that the risk of pre-diabetes mellitus (Pre DM) people developing diabetes is significantly increased, and about 5%-10% of Pre DM patients will turn into diabetic patients every year [5] [6] [7]. Pre-DM refers to a state in which the blood sugar level is higher than the normal range but has not yet reached the diagnostic standard of diabetes, which mainly includes Impaired Fasting Glucose (IFG) and Impaired Glucose Tolerance (IGT). In recent years, with changes in lifestyle and the ageing of the population, the incidence of pre-diabetes (Pre DM) has been rising, showing a trend of youthfulness. According to statistics, approximately one-third of adults worldwide are in a pre-diabetes state, and in China, this proportion is also high [8] [9]. In addition, Pre DM not only increases the risk of DM but also increases the risk of cardiovascular diseases, microangiopathy and other chronic diseases [10] [11]. Many studies have confirmed that PDM can also cause microvascular and macrovascular diseases, and its prevalence rate is significantly higher than that of people with normal glucose metabolism, which is clearly related to chronic hyperglycemia [12] [13]. At the same time, the prevalence rates of related complications, such as nerve damage, kidney disease, and retinopathy, were 11.2%, 16%, and 10% in the IGT population, and 3.9%, 4%, and 6.7% in the normal glucose metabolism population. Every time FPG increases by 1mmol/L, compared with people with normal glucose metabolism, the risk of cardiovascular disease in the PDM population will increase by 0.9 times [14] [15] [16].

Early intervention in the DM population can effectively delay or prevent its transformation to DM and reduce the risk of DM and its complications. Many studies, both at home and abroad, have confirmed the effectiveness of lifestyle interventions (such as a balanced diet, moderate exercise, and weight control) and drug interventions (such as using metformin) for the pre-diabetes (Pre DM) population [17] [18]. Because the Pre DM population usually has no obvious symptoms, how to screen this high-risk population in a timely and accurate manner has become a key research focus.

Due to their unique working environment and living conditions, the cadres' healthcare population frequently faces greater health challenges. Therefore, studying the risk factors of healthcare populations among pre-diabetic (Pre DM) cadres is helpful in formulating targeted intervention strategies and improving the health level of cadres. The purpose of this study is to identify the risk factors for diabetes mellitus (DM) in the pre-diabetes (Pre DM) cadres' healthcare population within 5 years and to provide a scientific basis for formulating effective intervention measures.

## Materials and methods

### Research object

**Figure 1:**
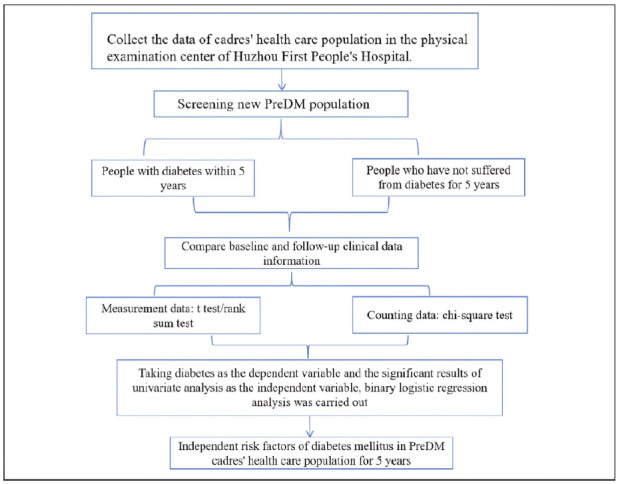
Technology roadmap.

In this study, the physical examination data from the physical examination centre of the First People's Hospital of Huzhou City from April 1, 2019, to November 1, 2024, were selected, and the cadres who had newly developed PDM and completed five-year follow-up were chosen as the research objects. The data, including gender, age, body mass index (BMI), and waist circumference, were collected and divided into two groups: diabetic and non-diabetic. The baseline data between the two groups were then compared. This study has obtained the informed consent of patients and the approval of the ethics Committee of Huzhou First People's Hospital. Figure 1

### Research object inclusion criteria

(1) Age ≥30 years old;

(2) The physical examination must have fasting blood glucose and glycosylated haemoglobin data;

(3) 6.1 ≤ fasting blood glucose < 7 mmol/L and/or 5.7%≤ glycosylated hemoglobin < 6.5%;

(4) No previous history of diabetes or high blood sugar;

(5) No follow-up within 5 years, and there must be physical examination data in the sixth year, and diabetes occurs within 5 years.

### Research object exclusion criteria

(1) Have a history of using hypoglycemic agents and steroid hormones within one year;

(2) Severe liver and kidney dysfunction;

(3) Severe anaemia, hemoglobinopathy, pregnancy, AIDS and malignant tumour;

(4) Recurrent acute pancreatitis, history of acute pancreatitis attack in recent 3 months, and history of operation in recent 3 months.

### Sample size calculation

According to the formula: n=Z^2\frac{P(1-p)}{E^2}, calculate the required sample size. Let the confidence level be 95%, the z value be 1.96, and the allowable error limit be 5% (E=0.05). In China, approximately 35.7% of the population is in the early stage of DM. For these people, there is a 5%-10% chance that they will further deteriorate into type 2 diabetes every year. In the long run, 33%-65% of pre-diabetic (Pre DM) patients will eventually progress to diabetes (DM) every year, so the sample size is 115. In this study, 174 cases of pre-diabetes cadres were finally included, which met the sample size requirements of this part of the study on influencing factors.

### Diagnostic criteria

The diagnostic criteria of Pre DM refer to the ADA clinical guidelines in 2010: IFG (5.6 mmol/L≤FPG<7.0 mmol/L) and/or IGT (7.8 mmol/L≤2h PG<11.1 mmol/L) and/or elevated HbA1c (5.7%≤HbA1c<6.5%) are defined as Pre DM. Because the data of this study comes from the physical examination data of the physical examination centre and lacks postprandial blood glucose, this study is defined according to the levels of fasting blood glucose (FPG) and/or glycosylated haemoglobin (HbA1c):

(1) Impaired fasting blood glucose (IFG): FPG is between 6.1 mmol/L and 6.9 mmol/L; that is, the fasting blood glucose value is greater than 6.1 mmol/L but less than 7.0 mmol/L.

(2) HbA1c): HbA1c is between 5.7% and 6.4%; that is, the HbA1c value is greater than 5.7% but less than 6.4%.

### Observation indicators

### Basic information

(1) Basic information: gender, age, height, weight, body mass index (BMI), waist circumference, marital status, nationality, job type, on-the-job status, etc.

(2) Vital signs: SBP DBF, pulse, etc.

(3) Past history: hypertension, diabetes, hyperglycemia, hyperlipidemia, etc.

(4) Smoking history

### Laboratory indicators

(1) Blood routine: white blood cells (WBC), red blood cells (RBC), haemoglobin (Hb), platelet count (PLT), etc. Sysmex XN-9000 automated haematology analyser was used for routine blood analysis, and the matching reagents provided by Sysmex were used. Standardised methods were used to determine all blood cell analysis indexes. The laboratory conducts indoor quality control every day. The quality control product is Bio-Rad Liquichek Chemistry Control, and the quality control results are all within the acceptable range.

(2) Blood lipid level: total cholesterol (TC), triglyceride (TG), high-density lipoprotein cholesterol (HDL-C) and low-density lipoprotein cholesterol (LDL-C). Roche Cobas c702 automatic biochemical analyser was used for blood lipid analysis, and the matching reagents provided by Roche Diagnostics were used. All blood lipid indexes were determined by standardised methods recommended by the International Federation of Clinical Chemistry (IFCC). The laboratory conducts indoor quality control daily, using Bio-Rad Liquichek Lipid Control as the quality control product. The quality control results are consistently within the acceptable range.

(3) Blood sugar level: fasting blood sugar (FPG) and glycosylated haemoglobin (HbA1c). Roche Cobas c702 automatic biochemical analyser was used for blood sugar analysis, and the matching reagents provided by Roche Diagnostics were used. Glycosylated haemoglobin was determined by high-performance liquid chromatography.

The laboratory conducts indoor quality control daily, using Bio-Rad Liquichek Diabetes Control as the quality control product. The quality control results are consistently within the acceptable range.

(4) Renal function: Serum uric acid level (SUA), serum creatinine level (Scr), urea nitrogen (BUN), creatinine (Cr), glomerular filtration rate (eGFR) and urinary protein (none, positive 1). Roche Cobas c702 automatic biochemical analyser was used for renal function indexes, and the matching reagents provided by Roche Diagnostics were used. The glomerular filtration rate (eGFR) was calculated by the CKD-EPI formula. The laboratory conducts indoor quality control every day, using Bio-Rad Liquid Chekurine Chemistry Control as the quality control product. The quality control results are all within the acceptable range. Urine protein was detected by the urine test paper method, and the results were divided into negative and positive 1+.

(5) Liver function: total bilirubin (TBIL), alanine aminotransferase (ALT), aspartate aminotransferase (AST), γ-glutamyltransferase (γ-GT), etc. Roche Cobas c702 automatic biochemical analyser was used for liver function indexes, and the matching reagents provided by Roche Diagnostics were used. The laboratory conducts indoor quality control every day, using Bio-Rad Liquichek Liver Control as the quality control product. The quality control results are all within the acceptable range [19].

### Statistical methods

SPSS26.0 was used for statistical analysis. In view of the possible data missing, the missing variables >25% are deleted, the continuous variables ≤25% meet the normal distribution and are filled with the mean, the ones that do not meet the normal distribution are filled with the median, and the classified variables are filled with the mode. The data of continuous variables conform to the normal distribution, which is expressed by the mean standard deviation (M±SD). In this study, dependent diabetes mellitus is a binary variable; therefore, the comparison between groups employs a two-sample t-test. Those that do not conform to the normal distribution are represented by median plus quartile interval, and the comparison between groups is based on the rank sum test of two independent samples in the non-parametric test. Then, the independent risk factors of pre-diabetes mellitus (Pre DM) in the cadre healthcare population were determined using logistic regression analysis, with diabetes occurrence as the dependent variable and statistically significant indicators from single-factor analysis as the independent variables. All statistical tests are bilateral probability tests, and P<0.05 is statistically significant.

## Results

### Pre-diabetes univariate analysis

The healthcare population of pre-diabetic (prediabetes, or Pre DM) individuals involved in this study has specific demographic characteristics. In terms of gender distribution, the non-DM group (n=144) consisted of 129 men and 15 women. In the DM group (n = 30), there were 24 men and 6 women. Finally, 174 cases of pre-diabetic (Pre DM) patients who met the admission and discharge criteria were included, of which 30 cases developed diabetes mellitus (DM), with an incidence rate of 17.2%. The non-parametric rank sum test revealed significant differences in age, sex, BMI, blood pressure, blood lipids, liver function, and renal function between the DM group and the non-DM group (P<0.05). See Table 1 for details.

**Table 1:** Univariate analysis of general data of DM group and DM group without DM.

Basic feature	No DM for 5 years<br>(n = 144)	DM within 5 years<br>(n=30)	*P*
Numerical variable			
Age (years)	60.96±12.11	69.2±12.78	<0.001
BMI (kg/m^2^)	22.67±2.68	26.34±2.11	<0.001
SBP (mmHg)	134.12±16.52	136.9±18.91	0.017
DBP (mmHg)	80.79±11.82	74.37±13.35	<0.001
Pulse (bpm)	73.31±9.55	76.97±9.82	0.029
γ-GT (U/L)	28.37±10.45	37.73±16.46	<0.001
Total Protein (g/L)	71.46±4.76	71.19±4.22	0.988
Albumin (g/L)	43.9±2.29	44.0±1.92	0.054
TC (mmol/L)	4.23±0.89	5.7±0.52	<0.001
TG (mmol/L)	1.23±0.54	2.98±2.45	0.003
HGB (g/L)	147.88±15.01	144.43±16.2	0.051
WBC (x10^9^/L)	6.22±1.54	6.19±1.63	0.072
RBC (x101^2^/L)	4.78±0.52	4.7±0.54	0.001
SUA (μmol/L)	333.84±85.9	453.67±64.28	<0.001
Cr (μmol/L)	77.75±12.86	131.87±18.12	<0.001
TBIL (μmol/L)	14.22±8.33	23.35±5.93	<0.001
AST (U/L)	19.87±4.79	23.6±6.62	0.004
ALT (U/L)	22.01±10.22	28.6±12.55	<0.001
PLT (x10^9^/L)	212.47±54.64	190.3±49.73	0.499
HbA1c(%)	6.05±0.21	6.42±0.29	<0.001
FPG (mmol/L)	5.66±0.55	6.51±0.94	<0.001
Classified variable			
Sex (Percentage)			
Male (%)	89.6	82.1	
Female (%)	10.4	17.8	

### Results of multivariate logistic regression analysis

Based on the results of univariate analysis, age, blood pressure, BMI, uric acid, creatinine, ALT and TG were taken as independent variables, and the occurrence of DM was taken as dependent variable to be analysed in the binary Logistic regression model. The results of the logistic regression analysis revealed that these 10 indicators were closely related to the occurrence of DM, with statistically significant differences (P<0.05). Table 2

**Table 2:** Multivariate analysis of general data of DM group and non-DM group norm.

	β	OR (95%CI)	P
Age	-0.002	0.99 (0.93, 1.064)	<0.001
BMI (kg/m^2^)	0.040	1.76 (1.02, 1.41)	<0.001
SBP (mmHg)	0.025	0.94 (0.85, 1.05)	<0.001
DBP (mmHg)	-0.097	0.90 (0.83, 0.99)	<0.001
Pulse (bpm)	0.072	1.08 (1.01, 1.14)	<0.001
γ-GT (U/L)	-0.012	1.06 (1.02, 1.09)	<0.001
TC (mmol/L)	2.259	9.57 (4.15, 22.02)	<0.001
TG (mmol/L)	3.192	24.35 (8.16, 72.59)	<0.001
FPG (mmol/L)	0.141	1.80 (1.04, 3.11)	<0.001
SUA (μmol/L)	0.194	1.02 (1.01, 1.02)	<0.001
AST (U/L)	0.060	1.06 (0.97, 1.16)	0.003
ALT (U/L)	0.027	1.03 (0.98, 1.07)	0.049
HbA1c (%)	1.124	3.06 (1.02, 9.18)	<0.001

## Discussion

Pre-diabetes is a necessary process for developing diabetes. In China, DM has the characteristics of high morbidity, high mortality and high disability rate. According to the International Diabetes Federation's report, in 2021, the number of diabetic patients worldwide reached 537 million, and it is predicted to increase to 643 million by 2030 and 783 million by 2045 [20]. DM is incurable at present, which seriously threatens the health of our people and makes our public health face severe challenges. In this study, when we discuss the related factors of Pre DM developing into DM in cadres' health care population, we find that there are significant differences in key indexes such as age, gender, BMI (body mass index), blood pressure, blood lipid, liver function and renal function between the two groups.

With the increase of age, the function of human islet β cells gradually declines, and insulin resistance also increases. This study found that older pre-diabetic (Pre DM) cadres are more likely to develop diabetes, which is consistent with the conclusions of previous studies [21]. This phenomenon may be closely related to age-related physiological changes, such as a decrease in muscle mass, changes in adipose tissue distribution, and a decline in endocrine system function. It is worth noting that because of the nature of their work, cadres may be in a sedentary state for a long time and lack regular physical activity. With the increase of age, the negative impact of this unfavourable lifestyle on blood glucose metabolism will be more significant, accelerating the occurrence of diabetes. Therefore, we should pay special attention to the lifestyle intervention of older pre-diabetic (pre-diabetes, or Pre DM) individuals and encourage more physical activity to improve insulin sensitivity and delay the onset of diabetes.

Body mass index (BMI) is a crucial measure for assessing the severity of obesity. Obesity, especially central obesity characterised by increased waist circumference, is closely related to insulin resistance [22]. The results of this study show that the risk of developing diabetes in Pre DM cadres with higher BMI and larger waist circumference is significantly increased. The mechanism may be that excessive adipose tissue secretes a variety of pro-inflammatory factors and free fatty acids, which can interfere with the insulin signal transduction pathway, inhibit the biological effect of insulin, and lead to the aggravation of insulin resistance, ultimately promoting an increase in blood sugar levels [22]. In addition, adipose tissue may further aggravate insulin resistance by affecting the secretion of adiponectin and other hormones [23]. Therefore, controlling weight, especially reducing the accumulation of abdominal fat, is of great significance for preventing diabetes.

Triglyceride (TG) is an important component of blood lipids and a key index for evaluating cardiovascular risk. Hypertriglyceridemia is an important part of metabolic syndrome, which is closely related to insulin resistance, β cell dysfunction and the occurrence and development of diabetes [24] [25]. Hyperlipidemia, especially hypercholesterolemia, promotes an inflammatory reaction and intensifies insulin resistance, thereby affecting blood sugar stability. Studies have shown that diabetic patients are often accompanied by a disorder of glucose metabolism, which will further interfere with the balance of lipid metabolism. Specifically, insulin resistance or insufficient insulin secretion will lead to the body's enhanced catabolism of fat, which will increase the level of free fatty acids in the blood and then induce hyperlipidemia. Excessive accumulation of free fatty acids will further aggravate insulin resistance and form a vicious circle. In addition, the activity of lipoprotein lipase, which is responsible for breaking down and transporting lipoproteins in diabetic patients, may decrease, affecting the normal metabolism and transportation of blood lipids, and is more likely to lead to dyslipidemia, especially the significant increase in triglyceride levels. This interaction between hyperlipidemia and diabetes will not only aggravate insulin resistance but also worsen the condition of diabetes and increase the difficulty of treatment [26] [27]. More importantly, long-term dyslipidemia in diabetic patients will significantly increase the risk of various complications and seriously affect the quality of life and life expectancy of patients. For example, studies have found a significant positive correlation between hyperlipidemia and the occurrence and severity of diabetic retinopathy [28], suggesting that controlling blood lipids is crucial in preventing diabetic retinopathy. Therefore, in the comprehensive management of diabetes, we should not only focus on controlling blood sugar levels but also regulate blood lipids, thereby reducing the risk of complications and improving patients' overall health. The results of this study also show that high triglyceride is an independent risk factor for the development of diabetes in prediabetic cadres' health care population, suggesting that we should strengthen the blood lipid management of hypertriglyceridemia population, reduce triglyceride levels through diet control, exercise and drug treatment, improve insulin sensitivity and prevent the occurrence of diabetes.

Hypertension and diabetes often coexist, and they share a similar pathogenesis, including insulin resistance, obesity, inflammatory reactions, and genetic susceptibility [24]. Long-term hypertension can damage renal blood vessels, leading to impaired renal function. The kidney plays a vital role in regulating blood sugar [29]. In this study, an increase in blood pressure (SBP and DBP) is associated with the development of diabetes in pre-diabetic (pre-diabetes, or Pre DM) individuals. Patients with hypertension may experience vascular endothelial dysfunction, which can impact the transport and function of insulin. At the same time, long-term hypertension will also aggravate the damage of target organs, such as the heart, brain and kidney, and further affect blood glucose metabolism [30]. Therefore, in the pre-diabetes population, actively controlling blood pressure not only helps prevent cardiovascular diseases but also helps delay the onset of diabetes.

The liver and kidneys are important metabolic organs of the human body, and their abnormal functions can affect blood sugar metabolism. Abnormal liver function will lead to a decrease in glycogen synthesis and an increase in gluconeogenesis, resulting in elevated blood sugar levels. Abnormal renal function leads to a decreased insulin clearance rate and increased insulin resistance, thereby increasing the risk of diabetes. In addition, liver and kidney dysfunction may also interact with inflammatory reactions, oxidative stress, and other factors to jointly promote the occurrence of diabetes [31] [32] [33]. The results of this study show that blood urea nitrogen (BUN), total bilirubin (TBIL), and alanine aminotransferase (ALT) are independent risk factors for the progression from pre-diabetes mellitus (Pre DM) to diabetes, suggesting that abnormal liver and kidney function may play a significant role in the occurrence and development of diabetes. Therefore, in the pre-diabetic (Pre DM) population, we should closely monitor changes in liver and kidney function indexes and take timely intervention measures to protect liver and kidney function and prevent the onset of diabetes.

To summarise, a five-year retrospective study of the healthcare population of pre-diabetic (Pre DM) cadres revealed that older age, high BMI, high fasting plasma glucose (FPG), high triglycerides (TG), and lack of exercise were independent risk factors for this population to develop diabetes within five years. In view of these risk factors, comprehensive intervention measures should be taken, such as strengthening health education, advocating a healthy lifestyle (reasonable diet, moderate exercise, smoking cessation and alcohol restriction, etc.), conducting regular health checkups, focusing on the control of blood sugar, blood lipid, blood pressure and other indicators, and early screening and intervention of high-risk groups with family history, so as to reduce the risk of Pre DM cadres' health care population developing into diabetes and improve the health level of cadres.

However, this study has several limitations. Firstly, this is a single-centre retrospective study, which may limit the generalizability of the research results to other populations and environments. Although the sample size meets the calculation requirements, it is relatively small, which may increase the risk of Type II errors. Secondly, retrospective design relies on existing medical records, which may be incomplete or contain inaccurate information, potentially introducing bias. Thirdly, although we have collected data on several potential risk factors, there may be other unmeasurable confounding factors, such as genetic factors, socioeconomic status, or specific eating habits, that may affect the progression from pre-diabetes to diabetes. Fourthly, the research population consists of the healthcare workforce, which may not be representative of the general population, thereby limiting the external validity of the research results. Finally, the diagnostic criteria of pre-diabetes and diabetes may have changed during the five-year study, which may introduce some heterogeneity in the classification of participants. Therefore, future research should address these limitations by employing multicentre, prospective designs with larger samples and more comprehensive data collection on potential risk factors, as well as standardised diagnostic standards.

## Dodatak

### Conflict of interest statement

All the authors declare that they have no conflict of interest in this work.
